# Incretin analogues as cardiovascular agents: a state-of-the-art review

**DOI:** 10.3389/fendo.2026.1898812

**Published:** 2026-07-24

**Authors:** Diego Araiza-Garaygordobil, Jorge Rico-Fontalvo, Betsabe Hernández-Balbuena, Monica Vinay-Coro, Mauricio González-Arias

**Affiliations:** 1 Departamento de Urgencias y Unidad Coronaria, Instituto Nacional de Cardiología Ignacio Chávez, Tlalpan, Ciudad de México, Mexico; 2 División de Estudios de Posgrado, Facultad de Medicina, Universidad Nacional Autónoma de México, Coyoacán, Ciudad de México, Mexico; 3 Comité de Riñón, diabetes y metabolismo, Asociación Colombiana de Nefrología e HTA, Bogotá, Colombia; 4 Department of Nephrology, Nephromedicall Health Services Provider Institution (IPS), Medellín, Colombia; 5 Department of Endocrinology, Hospital Center, New York, NY, United States

**Keywords:** cardiovascular outcomes, GLP-1 receptor agonists, heart failure with preserved ejection fraction, low- and middle-income countries, obesity, semaglutide, tirzepatide, type 2 diabetes mellitus

## Abstract

Glucagon-like peptide-1 (GLP-1) receptor agonists and the dual glucose-dependent insulinotropic polypeptide (GIP) and GLP-1 receptor agonist tirzepatide have evolved over the past decade from glucose-lowering antidiabetic agents into a cardiovascular drug class with proven outcome benefit across multiple clinical scenarios. This state-of-the-art review synthesizes the cardiovascular evidence base for incretin analogues, organized by clinical scenario, and addresses the structural challenges that constrain their deployment in low- and middle-income countries (LMICs). We review the pharmacology and proposed cardiovascular mechanisms, including direct effects on the vascular endothelium, macrophage inflammation, and epicardial adipose tissue, alongside indirect benefits mediated by weight loss, glycemic control, and blood pressure reduction. We summarize the evidence in five clinical scenarios: type 2 diabetes with established atherosclerotic cardiovascular disease or high cardiovascular risk; overweight or obesity with established cardiovascular disease but without diabetes; obesity-related heart failure with preserved ejection fraction; chronic kidney disease; and symptomatic peripheral artery disease. Pipeline agents (retatrutide, CagriSema, survodutide, orforglipron, zenagamtide, and MariTide) are reviewed alongside their ongoing cardiovascular outcome trials. A dedicated section examines the global access perspective, including the disproportionately high burden of cardiometabolic disease in LMICs, the limited representation of regional populations in pivotal trials, and the structural barriers of cost and reimbursement that constrain access. We close with a practical algorithm for the clinician, an honest assessment of evidence gaps, and a calibrated outlook on the next generation of incretin-based cardiovascular therapy.

## 1 Introduction

Cardiovascular disease remains the leading cause of premature mortality worldwide, with a disproportionate share of disability-adjusted life years now occurring in low- and middle-income countries (LMICs), where the cardiometabolic transition is unfolding most rapidly. Large population-based surveys and global meta-analyses from Latin America, Asia, the Middle East, and parts of Africa consistently demonstrate a high and rapidly rising burden of dysglycemia, with type 2 diabetes affecting a substantial proportion of adults in many high-burden settings and dysglycemia (including diabetes and prediabetes) affecting approximately one-third of the population in several regions. In parallel, overweight and obesity are highly prevalent and continue to increase, affecting more than half of adults in many LMIC populations and exceeding 30% for obesity alone in multiple regions (1, 2). This convergence of metabolic and atherosclerotic disease defines a population at extraordinarily high cardiovascular risk and at the same time exposes the limits of conventional secondary prevention. Despite optimal use of statins, antiplatelet agents, renin–angiotensin–aldosterone system blockers, and contemporary antihyperglycemics, residual risk in patients with type 2 diabetes and established atherosclerotic cardiovascular disease remains substantial (3).

Over the past decade, the field has witnessed a decisive shift. Several drug classes originally developed for glycemic control or weight reduction have demonstrated, in adequately powered cardiovascular outcome trials, the capacity to reduce major adverse cardiovascular events (MACE), heart failure hospitalization, and progression of chronic kidney disease — effects that extend well beyond what their metabolic actions alone would predict (4). The clearest example is the family of incretin analogues. From the first dedicated cardiovascular safety trial of a glucagon-like peptide-1 receptor agonist (ELIXA, 2015), through the practice-changing demonstrations of MACE reduction with liraglutide (LEADER), semaglutide (SUSTAIN-6, SELECT, SOUL), albiglutide (HARMONY OUTCOMES), dulaglutide (REWIND), and efpeglenatide (AMPLITUDE-O), to the more recent expansion into heart failure with preserved ejection fraction (STEP-HFpEF, SUMMIT) and chronic kidney disease (FLOW), the cumulative evidence has transformed these molecules from incidentally cardioprotective antidiabetics into a cardiovascular therapy in their own right (5–15). The dual glucose-dependent insulinotropic polypeptide and GLP-1 receptor agonist tirzepatide, the first member of a new generation of multi-receptor incretin-based agents, has now reproduced this benefit in the largest active-comparator outcomes trial of the class (SURPASS-CVOT, 2025) (16).

For the practising clinician, three questions follow. First, what is the cardiovascular mechanism of action of these agents, and how much of the observed benefit is plausibly direct as opposed to mediated by weight loss, glycemic control, or blood-pressure lowering? Second, in which clinical scenarios is the evidence robust enough to inform routine practice? Third, given that the populations most likely to benefit reside in countries with constrained healthcare resources, how do cost, access, and the under-representation of LMIC populations in pivotal trials affect the translation of this evidence into care?

**Figure 1:**
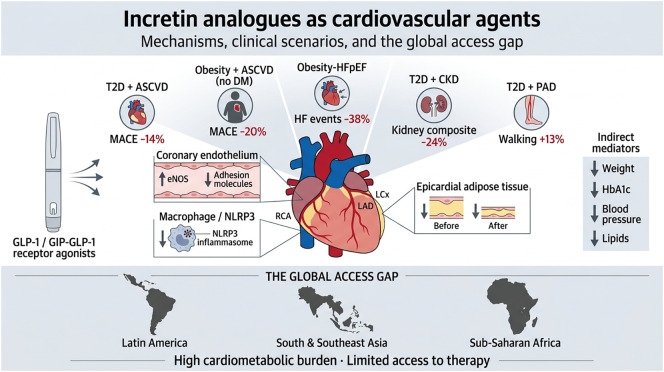
Central illustration of cardiovascular and cardiorenal benefits of incretin-based therapies across major clinical scenarios. GLP-1 receptor agonists and dual GIP/GLP-1 receptor agonists, including tirzepatide, have demonstrated cardiovascular and renal benefits in patients with type 2 diabetes, obesity, heart failure with preserved ejection fraction, chronic kidney disease, and peripheral artery disease. The figure summarizes the principal outcome trials and highlights reductions in major adverse cardiovascular events (MACE), heart-failure events, renal disease progression, and cardiovascular mortality, while emphasizing the global access challenges affecting low- and middle-income countries.

This narrative review addresses these questions, organized by clinical scenario rather than by drug. To facilitate navigation, Section 2 summarizes the cardiovascular mechanisms of incretin therapy, Sections 3–7 review the evidence across the major clinical scenarios, and Section 9 addresses implementation, access, and equity considerations in low- and middle-income countries. The agents reviewed include all approved GLP-1 receptor agonists (lixisenatide, liraglutide, exenatide, albiglutide, dulaglutide, semaglutide [subcutaneous and oral], efpeglenatide), the dual GIP/GLP-1 agonist tirzepatide, and the most advanced pipeline molecules with cardiovascular outcome trials underway (**Figure 1**).

## 2 Methodology

A narrative literature review was conducted to synthesize the current evidence on the cardiovascular effects of glucagon-like peptide-1 receptor agonists (GLP-1RAs) and the dual glucose-dependent insulinotropic polypeptide (GIP)/GLP-1 receptor agonist tirzepatide across different clinical settings.

The literature search was performed using PubMed/MEDLINE, Embase, and Google Scholar, focusing primarily on studies published between 2015 and 2026, while also including landmark studies and the most recent clinical practice guidelines from the European Society of Cardiology (ESC) and the American Diabetes Association (ADA).

Eligible publications included randomized controlled trials, cardiovascular outcome trials (CVOTs), systematic reviews, meta-analyses, mechanistic studies, and clinical practice guidelines. The evidence was organized into five predefined clinical scenarios: type 2 diabetes with established cardiovascular disease or high cardiovascular risk, obesity with established cardiovascular disease without diabetes, obesity-related heart failure with preserved ejection fraction, chronic kidney disease, and symptomatic peripheral artery disease. In addition, evidence regarding the mechanisms of cardiovascular protection, emerging incretin-based therapies, and the main barriers to their implementation in low- and middle-income countries was reviewed.

## 3 Pharmacology and cardiovascular mechanisms

### 3.1 The incretin axis and its receptors

GLP-1 is a 30–31 amino-acid peptide secreted by intestinal L cells in response to nutrient ingestion. Its physiological actions (glucose-dependent stimulation of insulin secretion, suppression of glucagon, delayed gastric emptying, and promotion of satiety) are mediated by the GLP-1 receptor, a class B G protein-coupled receptor expressed on pancreatic beta cells, neurons in the hypothalamus and brainstem, gastric smooth muscle, vascular endothelium, cardiomyocytes, vascular smooth muscle cells, and several immune lineages (17). The endogenous peptide is rapidly degraded by dipeptidyl peptidase-4, giving it a half-life of just a few minutes; therapeutic GLP-1 receptor agonists are engineered analogues with extended pharmacokinetics, ranging from once daily (liraglutide, oral semaglutide, lixisenatide) to once weekly (subcutaneous semaglutide, dulaglutide, exenatide extended-release, albiglutide, efpeglenatide) (18).

Glucose-dependent insulinotropic polypeptide (GIP) is a 42-amino-acid peptide secreted by intestinal K cells with complementary insulinotropic actions. Tirzepatide, a synthetic 39-amino-acid peptide based on the native GIP scaffold, activates both receptors with intrinsic bias toward GIP, producing additive metabolic effects that translate into greater glycemic and weight-loss efficacy than selective GLP-1R agonism (19). The pipeline now extends further: retatrutide adds glucagon receptor agonism (triple agonism), and survodutide is a dual glucagon/GLP-1 agonist (20).

### 3.2 Indirect cardiovascular effects

The cardiovascular benefit of GLP-1 receptor agonists is in part attributable to favorable changes in conventional risk factors. Across the pivotal trials, semaglutide, liraglutide, and dulaglutide consistently lower glycated hemoglobin by 0.7–1.5 percentage points, body weight by 3–15%, systolic blood pressure by 2–5 mmHg, and triglyceride and LDL-cholesterol levels by smaller but reproducible amounts. Tirzepatide produces larger reductions in weight (15–22%) and proportionally greater improvements in lipids, blood pressure, and glycemic control (21). Mediation analyses, however, have been instructive. In LEADER and REWIND, statistical modeling has attributed 30–40% of the MACE reduction to changes in glycated hemoglobin and urinary albumin–creatinine ratio, with smaller contributions from weight, blood pressure, and lipids; the majority of the cardiovascular effect is therefore not fully explained by changes in these surrogates (20, 21). Notably, in SELECT, which enrolled patients without diabetes, the curves for the primary cardiovascular endpoint began to separate within months of randomization — well before any meaningful weight loss had accrued — suggesting that mechanisms beyond weight reduction are operative (9).

### 3.3 Direct vascular and myocardial effects

**Figure 2:**
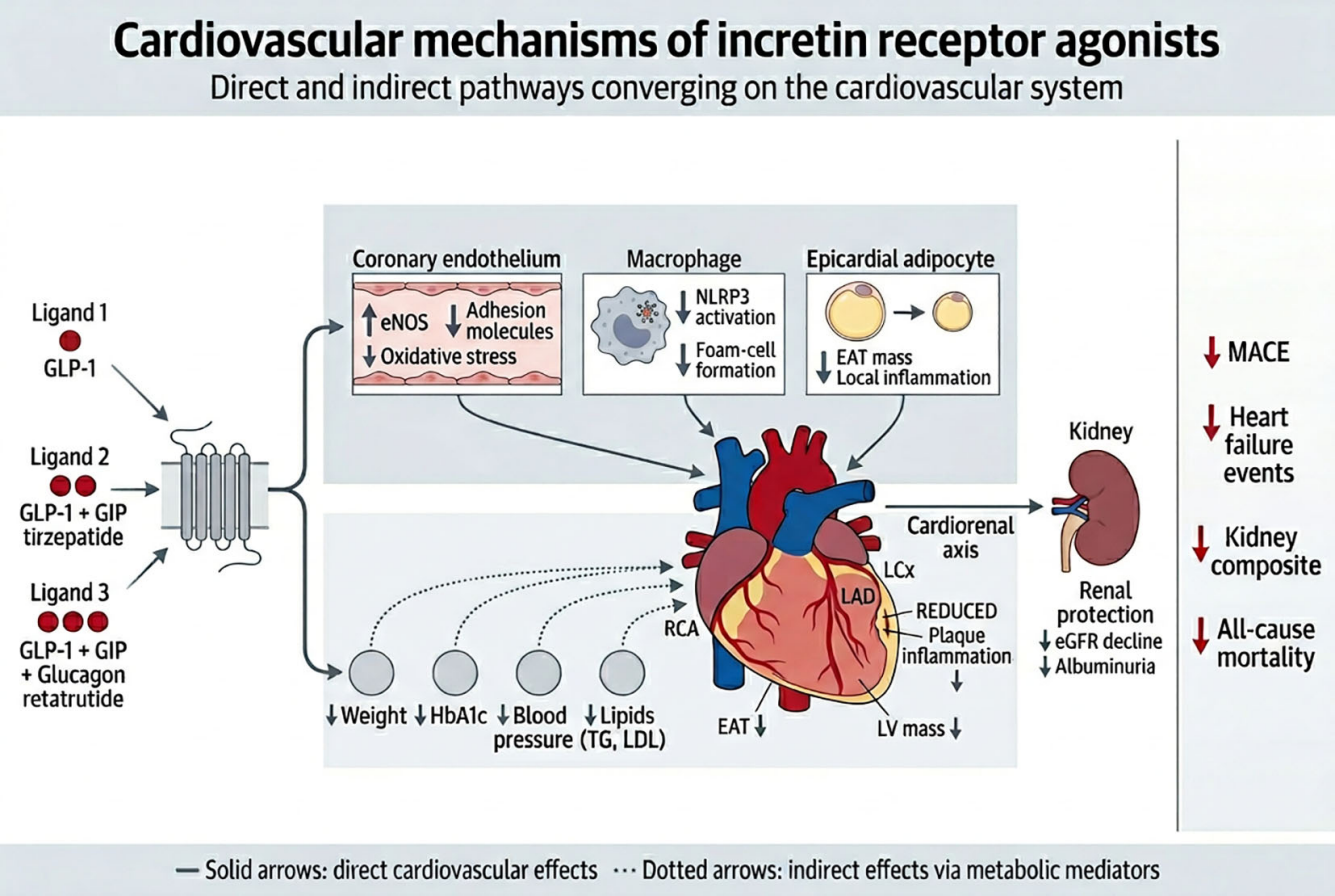
Proposed cardiovascular mechanisms of action of incretin-based therapies. Activation of GLP-1 and GIP/GLP-1 receptors exerts direct vascular, myocardial, inflammatory, and hemodynamic effects that extend beyond glycemic control. Proposed mechanisms include improvement of endothelial function, reduction of oxidative stress and inflammatory signaling, attenuation of macrophage activation and foam-cell formation, reduction of epicardial adipose tissue, improvement of myocardial energetics, and favorable metabolic and hemodynamic changes. Together, these effects contribute to reduced cardiovascular risk and improved cardiorenal outcomes.

GLP-1 receptors on the vascular endothelium, on macrophages, and on cardiomyocytes provide a plausible substrate for direct cardiovascular effects (**Figure 2**). In preclinical and translational studies, GLP-1 receptor activation increases endothelial nitric oxide synthase activity, suppresses the expression of vascular adhesion molecules, attenuates oxidative stress, inhibits the NLRP3 inflammasome in macrophages, and reduces foam-cell formation in atherosclerotic lesions (16–26). Imaging substudies in humans have shown reductions in coronary plaque volume and in markers of plaque inflammation with semaglutide and liraglutide, although these findings remain hypothesis-generating (27).

Inflammation appears to be a major unifying mechanism underlying these cardiovascular benefits. The reduction in high-sensitivity C-reactive protein (hsCRP) observed in STEP-HFpEF and SELECT (of the order of 42%) is one of the largest reported with any cardiometabolic drug class, and it correlates only loosely with weight loss (9, 14, 28). This is consistent with an anti-inflammatory action that may operate through direct receptor-mediated effects on macrophages and endothelial cells, as well as indirectly through reductions in adipose tissue mass and remodeling of visceral and epicardial fat depots. Epicardial adipose tissue thickness is rapidly and substantially reduced, reaching 29% at 3 months and 36% at 6 months with liraglutide in patients with type 2 diabetes and obesity. This reduction in inflammatory fat tissue is associated with a significant decrease in left ventricular mass, which could mediate the drug’s cardioprotective effects beyond glycemic control (29).

### 3.4 Heart failure–specific mechanisms

In obesity-related HFpEF, tirzepatide and semaglutide produce a constellation of effects that are mechanistically coherent with the proposed pathophysiology of this phenotype: reduction in plasma volume and circulatory overload, decrease in NT-proBNP and hsCRP, lowering of estimated left ventricular filling pressures, improvement in exercise capacity and quality of life, and a reduction in heart-failure events (30, 31).

### 3.5 Implications for the clinician

Three corollaries follow. First, the effect of these agents on cardiovascular outcomes is only partially captured by changes in glycemia or weight; therefore, dose adjustment and treatment continuation should not be guided by glycemic targets alone. Second, the early separation of event curves in SELECT and FLOW suggests that the antiatherosclerotic and anti-inflammatory components of the effect emerge rapidly. Third, the convergence of mechanisms with those of SGLT2 inhibitors — anti-inflammatory action, reduction in epicardial adipose tissue, and renal protection — provides the biological rationale for the additive benefit observed when these classes are combined (32).

## 4 Type 2 diabetes with established atherosclerotic cardiovascular disease or high cardiovascular risk

This is the scenario in which the cardiovascular case for incretin analogues is most mature. Eleven dedicated cardiovascular outcome trials, enrolling more than 95,000 patients with type 2 diabetes, now constitute the foundational evidence (**Table 1**).

**Table 1:** Pivotal cardiovascular outcome trials of GLP-1 receptor agonists and dual incretin agonists.

Trial	Drug	Population	n	Median follow-up	Primary endpoint	HR (95% CI)
ELIXA	Lixisenatide	T2D + recent ACS	6,068	25 months	CV death, MI, stroke, UA hosp.	1.02 (0.89–1.17) — non-inferior
LEADER	Liraglutide	T2D + high CV risk (81% CVD)	9,340	3.8 years	3-point MACE	0.87 (0.78–0.97); P = 0.01
SUSTAIN-6	Semaglutide SC	T2D + high CV risk	3,297	2.1 years	3-point MACE	0.74 (0.58–0.95); P = 0.02
EXSCEL	Exenatide weekly	T2D ± CVD (73% with CVD)	14,752	3.2 years	3-point MACE	0.91 (0.83–1.00); non-inferior
HARMONY OUTCOMES	Albiglutide	T2D + established CVD	9,463	1.6 years	3-point MACE	0.78 (0.68–0.90); P = 0.0006
REWIND	Dulaglutide	T2D + CV risk factors or CVD (31% CVD)	9,901	5.4 years	3-point MACE	0.88 (0.79–0.99); P = 0.026
PIONEER 6	Oral semaglutide	T2D + high CV risk	3,183	15.9 months	3-point MACE	0.79 (0.57–1.11); non-inferior
AMPLITUDE-O	Efpeglenatide	T2D + CVD or CKD + risk factor	4,076	1.81 years	3-point MACE	0.73 (0.58–0.92); P = 0.007
STEP-HFpEF	Semaglutide 2.4 mg	Obesity-related HFpEF (no DM)	529	52 weeks	KCCQ-CSS and Δ weight	KCCQ +7.8; weight –10.7%
STEP-HFpEF DM	Semaglutide 2.4 mg	Obesity-related HFpEF + T2D	616	52 weeks	KCCQ-CSS and Δ weight	KCCQ +7.3; weight –6.4%
SELECT	Semaglutide 2.4 mg	Overweight/obesity + CVD (no DM)	17,604	39.8 months	3-point MACE	0.80 (0.72–0.90); P<0.001
FLOW	Semaglutide 1.0 mg	T2D + CKD	3,533	3.4 years	Kidney composite + CV death	0.76 (0.66–0.88); P = 0.0003
SUMMIT	Tirzepatide	Obesity-related HFpEF (LVEF ≥50%)	731	104 weeks	CV death or worsening HF; KCCQ	0.62 (0.41–0.95); KCCQ + 6.9
SOUL	Oral semaglutide	T2D + ASCVD and/or CKD	9,650	49.5 months	3-point MACE	0.86 (0.77–0.96); P = 0.006
SURPASS-CVOT	Tirzepatide vs dulaglutide	T2D + ASCVD	13,299	4 years	3-point MACE	0.92 (0.83–1.01); non-inferior

STEP-HFpEF and SUMMIT primary endpoints are not 3-point MACE; they reflect heart-failure-specific composites. SELECT enrolled patients without diabetes. SURPASS-CVOT used dulaglutide as active comparator. All trials prespecified independently adjudicated cardiovascular endpoints. ACS, acute coronary syndrome; ASCVD, atherosclerotic cardiovascular disease; CKD, chronic kidney disease; CI, confidence interval; CV, cardiovascular; CVD, cardiovascular disease; DM, diabetes mellitus; HF, heart failure; HFpEF, heart failure with preserved ejection fraction; HR, hazard ratio; KCCQ-CSS, Kansas City Cardiomyopathy Questionnaire Clinical Summary Score; LVEF, left ventricular ejection fraction; MACE, major adverse cardiovascular events; MI, myocardial infarction; SC, subcutaneous; T2D, type 2 diabetes; UA, unstable angina.

### 4.1 From cardiovascular safety to cardiovascular benefit

The class entered the cardiovascular arena under regulatory pressure, after the rosiglitazone controversy (33). ELIXA, the first GLP-1 receptor agonist cardiovascular outcomes trial, enrolled 6,068 patients with type 2 diabetes and a recent acute coronary syndrome and showed a neutral primary composite (HR 1.02; 95% CI 0.89–1.17) over a median 25 months (5). The picture changed with LEADER (2016), in which liraglutide reduced 3-point MACE by 13% in 9,340 patients with high cardiovascular risk over 3.8 years (HR 0.87; 95% CI 0.78–0.97; P = 0.01 for superiority), with a parallel reduction in cardiovascular and all-cause mortality (6). SUSTAIN-6 showed that subcutaneous semaglutide reduced MACE by 26% in 3,297 patients over 2.1 years (HR 0.74; 95% CI 0.58–0.95) (7).

EXSCEL (2017) tempered the early optimism: in 14,752 patients enrolled in a broader population, once-weekly exenatide produced a 9% relative reduction in MACE, trending toward but not reaching statistical significance (HR 0.91; 95% CI 0.83–1.00; P = 0.06) (34). HARMONY OUTCOMES (2018) re-established the trajectory with the largest reported relative effect for a GLP-1 receptor agonist in this population: a 22% reduction in MACE with albiglutide over 1.6 years (HR 0.78; 95% CI 0.68–0.90) (8). REWIND (2019) demonstrated a 12% reduction in MACE with weekly dulaglutide over 5.4 years (HR 0.88; 95% CI 0.79–0.99) and was the first trial to show benefit in a primary-prevention-leaning population (12). AMPLITUDE-O (2021), with efpeglenatide, reproduced the class effect (HR 0.73; 95% CI 0.58–0.92) and showed that the benefit is preserved in patients receiving SGLT2 inhibitors at baseline (13).

### 4.2 The semaglutide oral program

PIONEER 6 was the cardiovascular safety trial of the first oral GLP-1 receptor agonist; in 3,183 patients followed for a median 15.9 months, oral semaglutide showed a hazard ratio for MACE of 0.79 (95% CI 0.57–1.11), consistent with a potential cardiovascular benefit, although the trial was not powered to demonstrate superiority for MACE as a standalone outcome (35). SOUL (2025) provided definitive superiority data: in 9,650 patients with type 2 diabetes and established atherosclerotic cardiovascular disease, chronic kidney disease, or both, oral semaglutide 14 mg daily reduced MACE by 14% over a median 49.5 months (HR 0.86; 95% CI 0.77–0.96; P = 0.006), with the benefit preserved in patients receiving SGLT2 inhibitors (11). The availability of an oral formulation has particular relevance for healthcare systems where the cold-chain storage requirements of injectable peptide therapies limit deployment outside referral centers.

### 4.3 SURPASS-CVOT and the dual-agonist era

SURPASS-CVOT (2025) is the largest and longest trial in the field: 13,299 patients with type 2 diabetes and established atherosclerotic cardiovascular disease randomized to tirzepatide or dulaglutide as active comparator over a median 4 years (16). Tirzepatide met the prespecified non-inferiority margin (HR 0.92; 95.3% CI 0.83–1.01) with an 8% relative reduction that did not reach statistical superiority, but exploratory analyses showed a 16% reduction in all-cause mortality and larger improvements in glycated hemoglobin, weight, blood pressure, and lipids than dulaglutide. The choice of an active comparator reflects the field’s recognition that placebo-controlled designs are no longer ethically tenable in this population.

### 4.4 Class effects and meta-analytic estimates

Across placebo-controlled cardiovascular outcome trials in type 2 diabetes, meta-analyses have produced consistent estimates: a 14% relative reduction in MACE (HR ~0.86), 13% reduction in cardiovascular mortality, 12% reduction in all-cause mortality, 11% reduction in heart-failure hospitalization, and 21% reduction in composite kidney outcomes (36). The benefit appears consistent across age, sex, baseline glycated hemoglobin, body mass index, and the presence or absence of established cardiovascular disease, and is largely independent of background SGLT2 inhibitor use (37). It should be acknowledged, however, that differences in trial blinding and the practical challenges associated with injectable therapies may introduce potential sources of bias. Although cardiovascular endpoints were adjudicated by independent blinded committees, these methodological considerations should be taken into account when interpreting the overall evidence.

### 4.5 Stroke as a distinguishing feature

A pattern that distinguishes GLP-1 receptor agonists from SGLT2 inhibitors deserves emphasis. Across cardiovascular outcome trials, GLP-1 receptor agonists consistently reduce MACE, although the contribution of individual components varies between studies. In several major trials, reductions in non-fatal stroke were as large as or greater than those observed for non-fatal myocardial infarction, whereas in others, including SOUL, the benefit was driven predominantly by reductions in myocardial infarction, with no significant effect on stroke. This contrasts with SGLT2 inhibitors, whose cardiovascular benefit is primarily mediated through reductions in heart-failure hospitalization and cardiovascular death. For clinicians managing patients with prior stroke, atrial fibrillation, or carotid disease, the relatively stronger cerebrovascular signal observed across the GLP-1 receptor agonist class remains clinically relevant, while acknowledging heterogeneity between individual trials.

### 4.6 Take-home

In a patient with type 2 diabetes and either established atherosclerotic cardiovascular disease or multiple risk factors, a GLP-1 receptor agonist with proven cardiovascular benefit (semaglutide subcutaneous or oral, liraglutide, dulaglutide) or tirzepatide should be considered as part of standard cardiovascular care, in addition to and not instead of optimal lipid-lowering, antihypertensive, antiplatelet, and SGLT2-inhibitor therapy. The decision should not depend on glycemic targets, since the cardiovascular benefit is largely independent of glycated hemoglobin reduction.

## 5 Obesity without diabetes

For decades, the cardiovascular evidence base in obesity rested almost exclusively on observational data and surrogate endpoints. Lifestyle interventions failed to demonstrate a reduction in cardiovascular events despite producing meaningful weight loss; bariatric surgery achieves both, but it remains a procedure for a small fraction of eligible patients. Pharmacotherapy, until 2023, lacked a single agent with proven cardiovascular benefit in this population.

### 5.1 Select

The SELECT trial changed this. In 17,604 patients aged 45 years or older with body mass index ≥27 kg/m² and established cardiovascular disease but without diabetes, weekly subcutaneous semaglutide 2.4 mg was compared with placebo over a mean 39.8 months (9). The primary 3-point MACE composite occurred in 6.5% of the semaglutide arm and 8.0% of the placebo arm (HR 0.80; 95% CI 0.72–0.90; P<0.001 for superiority). Three observations have particular cardiological relevance.

First, the cumulative incidence curves separated within the first six months of treatment, well before the body-weight curves had fully diverged. By the end of follow-up the mean weight reduction with semaglutide was 9.4% — substantial, but not in the bariatric range. The temporal mismatch argues that the protective mechanism is at least partly weight-independent.

Second, the composite kidney outcome was reduced by 22% (HR 0.78; 95% CI 0.63–0.96), and the magnitude of preservation of estimated glomerular filtration rate was greater in patients with baseline impairment (38). This is the first demonstration of a renoprotective effect of a pharmacological agent in patients with overweight or obesity but without diabetes.

Third, in the prespecified analysis of the 4,286 SELECT participants with prevalent heart failure at baseline, semaglutide reduced both MACE (HR 0.72; 95% CI 0.60–0.87) and a heart-failure composite (HR 0.79; 95% CI 0.64–0.98), with consistent benefit in patients with reduced and preserved ejection fraction (39). For patients with obesity-related heart failure with reduced ejection fraction (HFrEF), however, the available evidence remains more limited. Although the SELECT subgroup analysis suggests a potential benefit regardless of ejection fraction, dedicated randomized trials specifically evaluating patients with obesity-related HFrEF are still lacking.

### 5.2 Clinical context

In a patient with prior myocardial infarction, prior stroke, or symptomatic peripheral arterial disease and a body mass index ≥27 kg/m², semaglutide 2.4 mg has demonstrated a 20% relative reduction in major cardiovascular events on top of contemporary secondary prevention. Limitations are equally real: discontinuation due to gastrointestinal adverse events was 16.6% versus 8.2% with placebo, and cost is a fundamental barrier (Section 9). This dropout rate highlights that tolerability, especially nausea, vomiting, and diarrhea, is a significant barrier to sustained therapy in clinical practice.

Other GLP-1 receptor agonists are approved for chronic weight management but have not yet reported a cardiovascular outcome trial in the absence of diabetes; the cardiovascular evidence in obesity without diabetes remains specific to semaglutide 2.4 mg and should not be reflexively extrapolated to the class.

## 6 Heart failure: the obesity-related preserved ejection fraction phenotype

For many years, heart failure with preserved ejection fraction occupied an awkward space in the therapeutic landscape. The recognition that obesity drives a particular phenotype — characterized by plasma volume expansion, elevated left ventricular filling pressures, low-grade systemic inflammation, increased epicardial adipose tissue, and disproportionate symptom burden relative to natriuretic peptide concentrations — opened a therapeutic window for incretin-based therapy (40, 41).

### 6.1 The STEP-HFpEF program

STEP-HFpEF (2023) randomly assigned 529 patients with symptomatic heart failure (left ventricular ejection fraction ≥45%, body mass index ≥30 kg/m², KCCQ Clinical Summary Score <90), and without diabetes, to weekly subcutaneous semaglutide 2.4 mg or placebo for 52 weeks (14). Semaglutide produced a mean 16.6-point improvement in KCCQ-CSS versus 8.7 with placebo (estimated treatment difference 7.8; 95% CI 4.8–10.9; P<0.001), and a 13.3% weight reduction versus 2.6% (P<0.001). Confirmatory secondary endpoints — 6-minute walk distance, a hierarchical composite that included death and heart failure events, and high-sensitivity C-reactive protein — all favored semaglutide.

STEP-HFpEF DM (2024) reproduced these findings in 616 patients with the same phenotype but with concomitant type 2 diabetes (28). KCCQ-CSS improved by 13.7 points with semaglutide versus 6.4 with placebo (estimated treatment difference 7.3; 95% CI 4.1–10.4), and weight loss was 9.8% versus 3.4% — somewhat blunted compared with the non-diabetic cohort. The pooled analysis confirmed consistency of the effect across age, sex, body mass index, glycated hemoglobin, and SGLT2 inhibitor use (42). What STEP-HFpEF and STEP-HFpEF DM did not establish, by design, was an effect on hard outcomes; the trials were powered for symptom and weight endpoints, follow-up was limited to 52 weeks, and event counts were modest. SUMMIT filled this gap.

### 6.2 SUMMIT: hard outcomes with tirzepatide

SUMMIT (2025) enrolled 731 patients with NYHA class II–IV heart failure, left ventricular ejection fraction ≥50%, body mass index ≥30 kg/m², and one of the following: elevated natriuretic peptides, structural heart disease, elevated filling pressures, or a heart-failure decompensation in the prior 12 months (15). Follow-up extended to a median 104 weeks. Tirzepatide reduced the primary composite (cardiovascular death or worsening heart failure event) by 38% (HR 0.62; 95% CI 0.41–0.95; P = 0.026), driven primarily by a 46% reduction in worsening heart-failure events (HR 0.54; 95% CI 0.34–0.85). KCCQ-CSS improved by 6.9 points more than placebo (P<0.001), 6-minute walk distance increased, body weight fell by 11.6%, and high-sensitivity C-reactive protein decreased by approximately 37%. A mechanistic substudy linked these clinical benefits to reductions in estimated blood volume, systolic blood pressure, troponin T, and inflammation, with correlations between reductions in C-reactive protein and improvements in 6-minute walk distance (31). Notably, SUMMIT did not require elevated natriuretic peptides for entry — a methodological detail with clinical relevance: in routine practice, a normal NT-proBNP in a patient with obesity and dyspnea should not be interpreted as ruling out HFpEF.

Although SUMMIT is the largest trial dedicated to HFpEF for an incretin agent to date, its sample size is modest compared to the event-driven Cardi. Outcome trials or vascular studies and replication in larger cohorts are justified. Importantly, these findings apply specifically to the obesity-related HFpEF phenotype. Whether the cardiovascular and functional benefits of incretin-based therapies extend to patients with heart failure with reduced ejection fraction (HFrEF), or to HF phenotypes not primarily driven by obesity, remains uncertain because dedicated randomized outcome trials are lacking.

### 6.3 Heart failure beyond obesity-related HFpEF: current evidence and ongoing studies

Outside obesity-related HFpEF, the evidence base for GLP-1 receptor agonists remains considerably less mature. Although the SELECT heart-failure subgroup suggested cardiovascular benefit across patients with both preserved and reduced ejection fraction, these analyses were exploratory and were not powered for heart-failure-specific outcomes. Therefore, they should be interpreted as hypothesis-generating rather than definitive evidence (35). Similarly, FLOW demonstrated fewer heart-failure events in parallel with its renal and cardiovascular benefits (43). Across cardiovascular outcome trials, meta-analyses indicate a modest but consistent approximately 11% reduction in hospitalization for heart failure with GLP-1 receptor agonists, substantially smaller than the benefit observed with SGLT2 inhibitors (36).

Importantly, no dedicated cardiovascular outcome trial has yet demonstrated improved clinical outcomes in established HFrEF. Consequently, extrapolation of the HFpEF experience to HFrEF should be avoided. The ongoing FIT-HF trial (NCT06423599) is evaluating whether semaglutide-induced weight loss provides incremental improvements in functional capacity and cardiac remodeling compared with intensive dietary weight loss in patients with obesity and HFrEF. This and other ongoing studies will help determine whether the benefits observed in obesity-related HFpEF represent a phenotype-specific effect or can be generalized across the broader heart-failure spectrum.

### 6.4 Practical implications

In a patient with symptomatic HFpEF (LVEF ≥45%) and obesity (body mass index ≥30 kg/m²), with or without type 2 diabetes, semaglutide 2.4 mg or tirzepatide should be considered as part of the heart-failure treatment plan. Tirzepatide has the advantage of a dedicated outcome trial demonstrating reduction in heart-failure events; semaglutide has more cumulative evidence across additional cardiovascular and renal scenarios. In patients with obesity-related HFpEF, the combination of an incretin-based therapy, an SGLT2 inhibitor, and a non-steroidal mineralocorticoid receptor antagonist represents an emerging disease-modifying strategy supported by complementary mechanisms of action and an expanding evidence base. Whether a similar approach benefits patients with HFrEF remains unknown and awaits the results of ongoing dedicated clinical trials (44).

## 7 Chronic kidney disease and the cardiorenal axis

### 7.1 The FLOW trial

FLOW (2024) enrolled 3,533 patients with type 2 diabetes and chronic kidney disease, defined by an estimated glomerular filtration rate of 50–75 ml/min/1.73 m² with a urinary albumin-to-creatinine ratio of 300–5,000 mg/g, or an eGFR of 25–50 ml/min/1.73 m² with a UACR of 100–5,000 mg/g (10). All patients received background therapy with a renin–angiotensin system inhibitor at the maximum tolerated dose. They were randomly assigned to weekly subcutaneous semaglutide 1.0 mg or placebo. The primary composite outcome included onset of kidney failure, at least a 50% reduction in eGFR from baseline, or death from kidney-related or cardiovascular causes. The trial was stopped early for efficacy after a median 3.4 years.

Semaglutide reduced the primary outcome by 24% (HR 0.76; 95% CI 0.66–0.88; P = 0.0003), with each component contributing. The annualized rate of decline in estimated glomerular filtration rate was attenuated by 1.16 ml/min/1.73 m²/year—an effect of similar magnitude to that reported with SGLT2 inhibitors and finerenone in dedicated chronic kidney disease outcome trials (45, 46). The results were consistent across the entire spectrum of baseline kidney function and albuminuria (47).

### 7.2 Cardiovascular and survival outcomes in FLOW

The cardiovascular substudy of FLOW showed that the composite of cardiovascular death, non-fatal myocardial infarction, or non-fatal stroke was reduced by 18% (HR 0.82; 95% CI 0.68–0.98; P = 0.029), and all-cause mortality was reduced by 20% (HR 0.80; 95% CI 0.67–0.95; P = 0.01) (48). The mortality benefit was largest in patients with baseline UACR ≥300 mg/g — the population with the highest underlying cardiovascular risk. FLOW therefore provided, in a single trial, the most direct trial-level evidence to date of the bidirectional cardiorenal benefits of GLP-1 receptor agonism.

### 7.3 Take-home

For a patient with type 2 diabetes and chronic kidney disease (eGFR 25–75 ml/min/1.73 m² with significant albuminuria), semaglutide 1.0 mg subcutaneously weekly has level 1A evidence for reduction in kidney disease progression, cardiovascular events, and all-cause mortality, on top of standard background therapy that includes a renin–angiotensin system inhibitor and, where indicated, an SGLT2 inhibitor and finerenone.

## 8 Beyond mace: peripheral artery disease

STRIDE (2025), a phase 3b trial, randomly assigned 792 patients with type 2 diabetes and symptomatic peripheral artery disease (Fontaine stage IIa, ankle-brachial index ≤0.90 or toe-brachial index ≤0.70) to weekly subcutaneous semaglutide 1.0 mg or placebo for 52 weeks (49). Semaglutide produced a 13% relative improvement in maximum walking distance (estimated treatment ratio 1.13; 95% CI 1.06–1.21; P = 0.0004), with parallel improvements in pain-free walking distance and quality of life. The tolerability profile in STRIDE was consistent with previous semaglutide trials. Nevertheless, gastrointestinal adverse effects should be monitored in patients with peripheral artery disease, as they may compromise treatment adherence.

The magnitude of functional improvement is modest in absolute terms (median treatment difference of approximately 26 meters), but is among the few demonstrations of pharmacological improvement in functional capacity in this notoriously refractory population. However, it remains uncertain whether this functional improvement translates into clinically meaningful reductions in limb events, amputations, or other patient-centered outcomes, as these endpoints were not evaluated in the STRIDE trial.

## 9 The pipeline

The cardiovascular evidence base for incretin therapy is being extended in three directions: more potent dual and triple agonists, oral formulations, and combination peptides. Retatrutide, a triple agonist of GLP-1, GIP, and glucagon receptors, has shown weight reductions of up to 24% in phase 2 (20); TRIUMPH-Outcomes, a dedicated cardiovascular outcomes trial in approximately 10,000 adults, will report in 2027–2028. CagriSema, a fixed-dose combination of cagrilintide and semaglutide, has shown weight reductions of approximately 20% in phase 3, with REDEFINE-3 evaluating cardiovascular outcomes and REDEFINE-4 directly comparing CagriSema with tirzepatide. Survodutide, a dual glucagon/GLP-1 agonist; orforglipron, a non-peptide oral GLP-1 receptor agonist with no fasting requirement, for which phase 3 cardiovascular outcome trial results are currently pending (ACHIEVE program); and MariTide, a long-acting GIP-receptor antagonist/GLP-1 agonist administered monthly. Unlike tirzepatide, which acts as a GIP receptor agonist, MariTide incorporates GIP receptor antagonism, a mechanistically distinct approach that may result in a different efficacy and tolerability profile, are all in phase 3 cardiovascular and metabolic outcome programs. Zenagamtide (formerly amycretin), a unimolecular GLP-1 and amylin receptor agonist developed by Novo Nordisk in both subcutaneous and oral formulations, demonstrated in phase 1b/2a body-weight reductions of up to 22–24% over 36 weeks with the subcutaneous formulation (50) and approximately 13% with the oral formulation over 12 weeks (51), and entered phase 3 development in 2026 (**Table 2**). The cumulative direction is greater efficacy, simpler administration, and broader indications. From a global access perspective, the development of effective non-peptide oral agents (such as orforglipron) is particularly relevant: small-molecule oral therapies are inherently easier and cheaper to manufacture, store, and distribute than injectable peptides, and may eventually narrow the access gap that currently characterizes this drug class.

**Table 2:** Selected pipeline incretin-based agents and ongoing cardiovascular outcome trials.

Agent	Mechanism	Pivotal trial	Population	n (target)	Primary endpoint	Expected results
Retatrutide	Triple agonist (GLP-1/GIP/glucagon)	TRIUMPH-3	BMI ≥27 + established CVD	~1,800	Body weight & CV biomarkers	2026
Retatrutide	Triple agonist (GLP-1/GIP/glucagon)	TRIUMPH-Outcomes	BMI ≥27 + ASCVD and/or CKD	~10,000	3-point MACE; kidney composite	2027–28
CagriSema	Cagrilintide + semaglutide	REDEFINE-3	BMI ≥27 + established CVD	~7,000	3-point MACE	2027
CagriSema	Cagrilintide + semaglutide	REDEFINE-4	Active comparator vs tirzepatide	~800	Body weight at week 72	2026
Survodutide	Dual agonist (glucagon/GLP-1)	Phase 3 CV program	Obesity + CVD or high CV risk	~5,000	3-point MACE	2028
Orforglipron	Oral non-peptide GLP-1 RA	ACHIEVE phase 3 program	T2D + CVD or high CV risk	~6,000	3-point MACE	2027–28
Zenagamtide (amycretin, NN 9487)	Unimolecular (GLP-1/amylin); SC and oral	REDEFINE (phase 3)	Obesity/T2D/HF	TBD	Body weight; CV biomarkers	2027–28
MariTide (maridebart cafraglutide)	GIP-R antagonist/GLP-1 RA (monthly)	Phase 3 CV program	Obesity + established CVD	~7,000	3-point MACE	2028–29

ASCVD, atherosclerotic cardiovascular disease; BMI, body mass index; CKD, chronic kidney disease; CV, cardiovascular; CVD, cardiovascular disease; GIP-R, glucose-dependent insulinotropic polypeptide receptor; GLP-1, glucagon-like peptide-1; HF, heart failure; MACE, major adverse cardiovascular events (CV death, non-fatal myocardial infarction, non-fatal stroke); RA, receptor agonist; SC, subcutaneous; T2D, type 2 diabetes mellitus; TBD, to be determined.

The FIT-HF trial (NCT06423599), an investigator-initiated phase II/III study of semaglutide 2.4 mg in patients with obesity and heart failure with reduced ejection fraction (n ≈ 100), is described in the main text given its distinct design relative to industry-sponsored pivotal outcomes trials.

Trial registration numbers, target enrolment, and projected completion dates reflect publicly available information at the time of search; tags marked [VERIFICAR] should be reconfirmed against clinicaltrials.gov before submission. Phase 3 obesity trials without cardiovascular outcome endpoints are excluded. ASCVD, atherosclerotic cardiovascular disease; BMI, body mass index; CKD, chronic kidney disease; CKM, cardiovascular-kidney-metabolic; CV, cardiovascular; CVD, cardiovascular disease; CVOT, cardiovascular outcome trial; GIP, glucose-dependent insulinotropic polypeptide; GLP-1, glucagon-like peptide-1; MACE, major adverse cardiovascular events; T2D, type 2 diabetes.

## 10 The global access perspective: incretin therapy in low- and middle-income countries

For all the abundance of evidence summarized above, its translation into routine practice is constrained by three structural realities that are particularly acute in LMICs: cost, availability, and the limited representation of regional populations in pivotal trials.

### 10.1 The disease burden is uniquely high

The case for incretin-based therapy in low- and middle-income countries (LMICs) is, in many respects, particularly compelling compared with high-income settings where the pivotal trials were conducted. Across regions including Latin America, South and Southeast Asia, the Middle East, North Africa, and parts of sub-Saharan Africa, large population-based studies and global meta-analyses consistently demonstrate a high and rapidly increasing burden of cardiometabolic disease. Type 2 diabetes affects a substantial proportion of adults in many high-burden populations, while dysglycemia (including diabetes and prediabetes) affects approximately one-third of individuals in several regions (52). In parallel, overweight and obesity are highly prevalent, affecting more than half of adults in many LMIC settings, with obesity alone exceeding 30% in multiple regions (1, 2). The cardiometabolic transition has occurred over a much shorter timeframe than in high-income countries, and frequently overlaps with persistent infectious disease and undernutrition burdens, generating a “double burden” health profile. Cardiovascular disease accounts for approximately one in four deaths in most of these settings. The population that meets the eligibility criteria for SELECT, FLOW, SUSTAIN-6, or SOUL is therefore enormous in absolute terms, and is growing.

### 10.2 Trial representation is limited

LMICs have been moderately represented in some pivotal trials and underrepresented in others. ELIXA and HARMONY OUTCOMES included substantial Latin American and Asia-Pacific sites; LEADER, REWIND, and SELECT included LMIC investigators and sites but in proportions that fall short of the regional disease burden. SUSTAIN-6 had limited LMIC participation. Detailed prespecified subgroup analyses by ethnicity or geography are sparse, and the adequacy of the pivotal evidence to populations of predominantly non-European ancestry — populations with documented differences in body composition, insulin resistance, and cardiovascular phenotype at any given body mass index — has not been formally tested. The biological plausibility of the class effect, however, makes broad generalizability the reasonable working assumption, and post-marketing real-world data from LMIC cohorts are accumulating (53).

### 10.3 Cost and access

The price of injectable incretin analogues remains the principal barrier to their use for cardiovascular indications in LMICs. As of early 2026, semaglutide for diabetes is available across many LMICs in a range of approximately USD 150–350 per pen, semaglutide 2.4 mg for weight management at USD 175–500 per dose depending on strength, and tirzepatide between USD 400–600 monthly (54). These figures, while substantially lower than United States list prices, remain prohibitive for the majority of patients without comprehensive private insurance. Public-sector reimbursement (national health insurance, social security systems) is typically restricted to specific endocrine indications — most commonly poorly controlled type 2 diabetes — with no current pathway for cardiovascular-only indication. Out-of-pocket expenditure for injectable incretin therapy can therefore consume a significant fraction of household income, even for middle-class patients in upper-middle-income countries.

The patent expiry of older agents in this class and the development of biosimilar liraglutide and semaglutide in regional manufacturing hubs — including India, Brazil, China, and Egypt — are expected to expand access progressively from the late 2020s onward. The advent of effective oral non-peptide GLP-1 agonists (Section 8), whose lower manufacturing cost and absence of cold-chain requirements are structurally favorable for resource-constrained settings, may further alter this landscape.

Beyond affordability, additional health-system barriers continue to limit implementation in many LMICs. These include the need for cold-chain infrastructure for injectable peptide therapies, shortages of healthcare professionals trained to initiate and monitor incretin-based treatment, and the absence of cardiovascular indications from national essential medicines lists and reimbursement policies in most countries. Addressing these structural barriers will be essential to achieve equitable access to these therapies.

### 10.4 Implications for the clinician in LMICs

The clinician’s role in this context becomes one of evidence-informed advocacy. The available data support the use of incretin analogues in well-defined, high-risk scenarios — established cardiovascular disease with type 2 diabetes, established cardiovascular disease with overweight or obesity without diabetes, obesity-related HFpEF, type 2 diabetes with chronic kidney disease — where the absolute benefit is largest. Patient selection should prioritize these scenarios, in dialogue with payers and patients about the realistic cost of treatment. International societies and regional cardiovascular networks have an important role to play in advocating for the inclusion of cardiovascular indications in essential medicines lists and reimbursement formularies.

## 11 Practical algorithm

For a patient with established atherosclerotic cardiovascular disease and type 2 diabetes, an incretin analogue with proven cardiovascular benefit (semaglutide, liraglutide, dulaglutide, or tirzepatide) should be considered as part of secondary prevention, irrespective of glycemic control. The combination with an SGLT2 inhibitor is supported by SOUL, AMPLITUDE-O, and FLOW substudies and is endorsed by current guidelines (4).

For a patient with established cardiovascular disease, overweight or obesity (BMI ≥27), and without diabetes, semaglutide 2.4 mg has level 1A evidence (SELECT) for cardiovascular event reduction.

For a patient with obesity-related HFpEF (LVEF ≥45%, BMI ≥30), with or without diabetes, semaglutide 2.4 mg or tirzepatide should be considered alongside an SGLT2 inhibitor and, where indicated, finerenone.

For a patient with type 2 diabetes and chronic kidney disease (eGFR 25–75 ml/min/1.73 m² with significant albuminuria), semaglutide 1.0 mg has level 1A evidence (FLOW) for reduction of kidney disease progression, cardiovascular events, and all-cause mortality.

For a patient with type 2 diabetes and symptomatic peripheral artery disease, semaglutide 1.0 mg may be considered to improve walking capacity, on the basis of STRIDE.

Across all scenarios, the principal contraindication is a personal or family history of medullary thyroid carcinoma or multiple endocrine neoplasia type 2; the principal practical issue is gastrointestinal tolerability, managed through gradual dose escalation. The cardiovascular benefit emerges within 3–6 months of initiation, supporting persistence at the maximum tolerated dose.

## 12 Limitations and evidence gaps

Several limitations of the current evidence deserve acknowledgement. Head-to-head trials between incretin analogues and SGLT2 inhibitors are absent, and the apparent additivity of the two classes derives from indirect comparisons. Long-term durability of the cardiovascular benefit beyond the trial follow-up periods (typically 2–5 years) remains to be established. Gastrointestinal-driven discontinuation rates in cardiovascular trials (15–25%) are substantially lower than in real-world practice, raising legitimate questions about the gap between efficacy and effectiveness. The cardiovascular evidence in heart failure with reduced ejection fraction outside the obesity phenotype is insufficient. Likewise, the benefit of incretin-based therapies in patients with heart failure with preserved ejection fraction and a normal body mass index (<25 kg/m²) has not been established, and the current evidence should not be extrapolated to this population. Although subgroup analyses have generally shown consistent relative risk reductions across sex and age groups, absolute treatment benefits are likely to vary according to baseline cardiovascular risk. In addition, adults older than 80 years remain underrepresented in most pivotal trials, limiting the applicability of the available evidence to this growing population. The representation of populations from LMICs — including Latin American, African, South Asian, and Southeast Asian populations — in pivotal trials remains modest, and the assumption of class effect across populations rests on biological plausibility rather than direct demonstration.

## 13 Conclusion

The accumulated evidence of the past decade has repositioned incretin-based therapies from glucose-lowering agents with established cardiovascular safety to therapies with proven cardiovascular benefit across atherosclerotic cardiovascular disease, obesity-related heart failure with preserved ejection fraction, and chronic kidney disease, with benefits observed in patients with and without type 2 diabetes depending on the clinical context. The next generation of agents, with greater efficacy and simpler administration (including oral non-peptide formulations of particular relevance for resource-constrained settings) is well advanced in clinical development. For clinicians practicing in LMICs, the principal challenges are no longer scientific but practical: identifying patients most likely to derive meaningful benefit, overcoming barriers to access, and integrating these therapies alongside established standards of cardiovascular and renal care. The disproportionate burden of cardiometabolic disease in LMICs means that these populations stand to benefit substantially from these advances, yet they remain among the least likely to receive them because of persistent structural barriers. Addressing this disparity will represent a major policy and implementation challenge for incretin-based cardiovascular medicine over the coming decade.
